# Decoding the role of exosomes in bladder cancer: focusing on tumor progression and immune microenvironment modulation

**DOI:** 10.3389/fonc.2026.1821922

**Published:** 2026-07-31

**Authors:** Xin Wang, ZhuYong Li, Bin Zhang

**Affiliations:** Department of Urology, Chengdu Seventh People’s Hospital (Affiliated Cancer Hospital of Chengdu Medical College), Chengdu, China

**Keywords:** biomarkers, bladder cancer, drug delivery, exosomes, immunotherapy, tumor microenvironment

## Abstract

Bladder cancer (BCa) remains one of the most prevalent urological malignancies worldwide and is characterized by high recurrence rates and substantial therapeutic challenges in advanced disease. Exosomes, a nanoscale subtype of extracellular vesicles, have emerged as important mediators of intercellular communication within the tumor microenvironment (TME). This review summarizes the roles of exosomes in BCa progression, immune microenvironment modulation, biomarker development, drug delivery, and immunotherapy, while critically distinguishing preclinical observations from early clinical biomarker evidence. BCa-associated exosomes can promote tumor growth, metastasis, angiogenesis, metabolic reprogramming, and chemoresistance by transferring bioactive cargoes, including microRNAs, long non-coding RNAs, proteins, lipids, and metabolites. Within the tumor immune microenvironment (TIME), BCa-derived exosomes contribute to immune evasion by promoting M2 macrophage polarization, impairing dendritic-cell function, and suppressing natural killer (NK)-cell and T-cell activity. To clarify the context-dependent effects of exosomes, we propose a cargo-context-recipient framework in which exosomal function is determined by the molecular cargo, the biological and therapeutic context, and the functional state of the recipient cell. Because urine provides direct access to bladder-derived vesicles, urinary exosomes show promise as non-invasive diagnostic and prognostic biomarkers, although most studies remain limited by small cohorts and require independent validation. Exosomes also represent potential drug-delivery and immunomodulatory platforms; however, bladder cancer-specific therapeutic evidence remains largely preclinical. Clinical translation is constrained by heterogeneity in exosome isolation and quantification, limited standardization, insufficient mechanistic validation, and a paucity of BCa-specific interventional trials. A more critical, mechanism-guided, and evidence-stratified framework is therefore needed to move exosome research in BCa from descriptive cargo cataloguing toward clinically actionable biomarkers and therapeutic strategies.

## 1 Introduction

Bladder cancer (BCa) is one of the most common urological malignancies, with more than 610,000 new cases and approximately 220,000 deaths reported worldwide in 2022 (1). Its prevalence is approximately three- to four-fold higher in men than in women, although the mechanisms underlying this sex disparity remain incompletely understood (2). Cigarette smoking and occupational or environmental exposure to toxic agents, including aniline dyes, remain major environmental risk factors (3). BCa encompasses a heterogeneous disease spectrum, ranging from recurrent non-muscle-invasive tumors requiring long-term surveillance to aggressive or advanced tumors requiring multimodal treatment. Approximately 75% of BCa cases are non-muscle-invasive at diagnosis (4, 5); however, progression to muscle-invasive BCa occurs in up to 20% of patients (6). Non-muscle-invasive bladder cancer (NMIBC) is associated with a relatively favorable prognosis but a high recurrence rate (7), whereas muscle-invasive bladder cancer (MIBC) carries a greater risk of metastasis and poor clinical outcomes (8). For patients with MIBC, treatment options remain limited, and the 5-year overall survival (OS) ranges from approximately 5% to 36% (9). Standard management typically combines surgery with chemotherapy, immunotherapy, or other multimodal strategies (10, 11). Nevertheless, primary treatment resistance, recurrence, progression, and metastasis remain major clinical challenges, underscoring the need to clarify the molecular mechanisms of BCa development and identify more effective therapeutic approaches (12).

Tumors are complex ecosystems in which the composition and functional state of the TME vary across cancer types and tumor-intrinsic phenotypes (13). Deciphering the interplay among tumor-intrinsic features, extrinsic microenvironmental cues, and systemic mediators of disease progression is essential for the rational design of anticancer therapies (14). Tumor cells are embedded within a dynamic microenvironment composed of innate and adaptive immune cells, vascular cells, cytokines, stromal cells, and extracellular matrix (ECM) components (15). These host-derived cells were once regarded as passive bystanders, but advances in single-cell sequencing and spatial transcriptomics have shifted the field from cataloguing cell types to dissecting intercellular communication networks (16, 17).

Exosomes are central mediators of TIME remodeling in BCa because they transfer molecular information among tumor cells, stromal cells, endothelial cells, and immune cells (18). As nanoscale vesicular messengers, exosomes deliver proteins, lipids, metabolites, and nucleic acids to recipient cells, thereby reshaping cellular phenotypes and functional states (19, 20). Through these cargoes, BCa-derived exosomes participate in immune escape, sustained proliferation, angiogenesis, metabolic rewiring, therapeutic resistance, and metastasis (21, 22). Elucidating the signaling pathways and molecular networks through which exosomes regulate the BCa TME may uncover clinically relevant vulnerabilities and guide the design of mechanism-based therapeutic strategies (23, 24). Owing to their low immunogenicity, limited inherent toxicity, natural targeting capacity, and excellent biocompatibility, exosomes have emerged as attractive delivery vehicles for antitumor agents in BCa, offering clear advantages over conventional drug carriers (25). In parallel, exosome-based, cell-free vaccine strategies have shown promising antitumor activity in preclinical and early clinical studies in other malignancies, highlighting their potential utility in bladder cancer immunotherapy (26). Strategically harnessing exosomes either as therapeutic targets or as engineered delivery systems may enhance and sustain antitumor immune responses within the TIME of BCa, making them a compelling and increasingly feasible avenue for future translational research and clinical application (18). In this review, we examine the complex roles of exosomes in BCa progression and immune modulation. We further summarize the mechanisms by which exosomes mediate crosstalk between BCa cells and the TME, with particular emphasis on their effects on cellular phenotypes and functions. Finally, we discuss the potential applications of exosome-based immunotherapies in BCa and outline future directions for the therapeutic development of exosome-based strategies. Compared with recent reviews that primarily catalogue exosomal cargoes, summarize extracellular vesicle biomarkers, or discuss exosome-based delivery in general cancer settings, this review makes three specific contributions. It integrates tumor progression and immune microenvironment modulation as interconnected processes rather than independent topics. Moreover, it explicitly stratifies the evidence according to experimental level, distinguishing *in vitro* findings, animal-model evidence, early clinical biomarker data, and findings requiring clinical validation. Furthermore, it develops a cargo-context-recipient framework to explain why the same class of exosomal cargo may exert divergent effects depending on tumor stage, cellular origin, treatment pressure, recipient-cell state, and microenvironmental context. This framework is intended to move BCa exosome research from descriptive molecular cataloguing toward mechanism-guided interpretation and clinically actionable hypotheses.

### 1.1 Literature search strategy

To improve methodological transparency, we conducted a structured literature search in PubMed, Web of Science, and Scopus for studies published up to January 5, 2026. Search terms included combinations of ‘bladder cancer’, ‘urothelial carcinoma’, ‘exosome’, ‘extracellular vesicle’, ‘tumor microenvironment’, ‘immune microenvironment’, ‘biomarker’, ‘drug delivery’, ‘immunotherapy’, ‘microRNA’, ‘lncRNA’, ‘chemoresistance’, and ‘angiogenesis’. English-language original research articles, reviews, and clinically relevant studies were considered eligible if they addressed exosome biogenesis, cargo function, BCa progression, immune modulation, diagnostic or prognostic biomarkers, or exosome-based therapeutic strategies. Conference abstracts, editorials without primary data, duplicate publications, non-English articles, and studies not directly relevant to exosome-related biology in BCa were excluded. As this is a narrative review rather than a systematic review, no formal meta-analysis was performed. Priority was given to studies that included mechanistic validation, human biofluid samples, independent validation cohorts, or clear relevance to BCa-specific biology (**Supplementary Table 1**).

## 2 The biogenesis and function of exosomes

**Figure 1:**
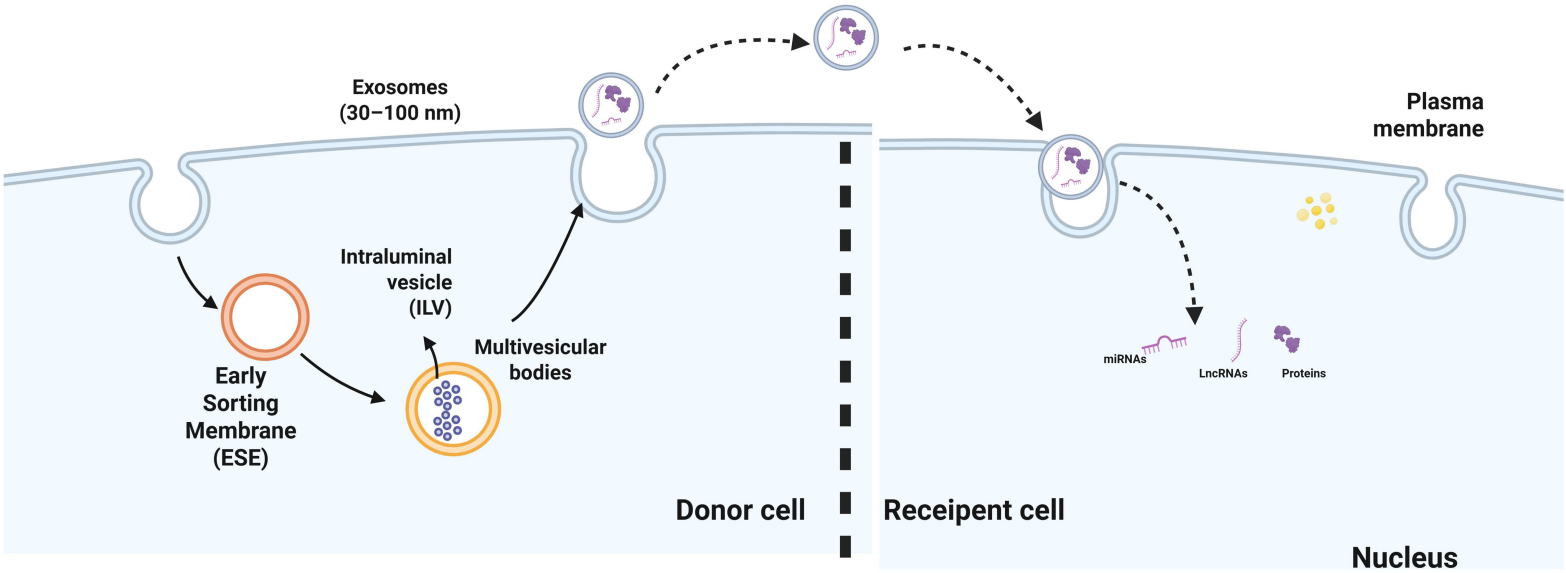
Biogenesis and cargo loading of BCa-derived exosomes. Exosomes originate from the endocytic pathway, where inward budding of early endosomes forms multivesicular bodies (MVBs). During this process, specific ncRNAs and proteins are sorted into intraluminal vesicles (ILVs) via Syntenin-1 and ESCRT-related machinery. Mature MVBs fuse with the plasma membrane to release exosomes into the extracellular matrix (ECM).

Exosomes are a nanoscale subtype of extracellular vesicles (EVs), typically 30–150 nm in diameter, and are important mediators of intercellular communication (27). Exosome biogenesis involves several coordinated steps (28). The process begins with invagination of the plasma membrane and incorporation of cell-surface and soluble extracellular molecules, resulting in the formation of early sorting endosomes (ESEs) (29). ESEs mature into late-sorting endosomes (LSEs), which subsequently form multivesicular bodies (MVBs) through inward budding of the endosomal limiting membrane (30). Consequently, MVBs contain multiple intraluminal vesicles (ILVs) (31). At this stage, MVBs can take one of two pathways: they may follow the lysosomal route and fuse with autophagosomes or lysosomes for degradation, or they may fuse with the plasma membrane, releasing the enclosed ILVs as exosomes (32, 33) (**Figure 1**).

Exosomes are released into the extracellular space when MVBs fuse with the plasma membrane (34). They are secreted by many cell types, including immune cells and cancer cells (35), and are detectable in multiple body fluids, including blood, plasma, urine, tears, and saliva (36, 37). Their cargoes include proteins, lipids, metabolites, and nucleic acids that partly reflect the biological state of the cells from which they originate (36). After reaching recipient cells, exosomes deliver their cargo through receptor-ligand interactions, direct membrane fusion, or endocytosis, with endocytosis being a common uptake route (38). Exosome uptake is not entirely random; recipient-cell receptors and adhesion molecules confer a degree of selectivity to cargo transfer, supporting the potential diagnostic and therapeutic utility of exosomes (39). Rather than engaging in completely random interactions with nearby cells, exosomes tend to be internalized by recipient cells that express compatible surface receptors and adhesion molecules. These receptor–ligand interactions confer a degree of selectivity to the transfer of their genetic cargo and underpin their value for diagnostic and therapeutic applications (40, 41).

Exosomes are involved in both cancer progression and therapeutic modulation (42, 43). They have been shown to influence key cellular events such as apoptosis, autophagy, metastasis and metabolic reprogramming (44–47). Through these diverse mechanisms, exosomes are likely to participate in cancer progression via multiple interconnected pathways. Additionally, through exosome-mediated intercellular interactions, they significantly contribute to immunoregulation, including processes such as antigen presentation, immune activation, immune suppression, and the establishment of immune tolerance (48, 49). However, their biological effects depend on vesicle biogenesis, cargo composition, donor-cell identity, recipient-cell state, and microenvironmental conditions (45, 50). This context dependence is particularly important in BCa, where exosome-mediated communication can have distinct effects across tumor stages, treatment settings, and immune states.

## 3 Role of exosomes in BCa

**Figure 2:**
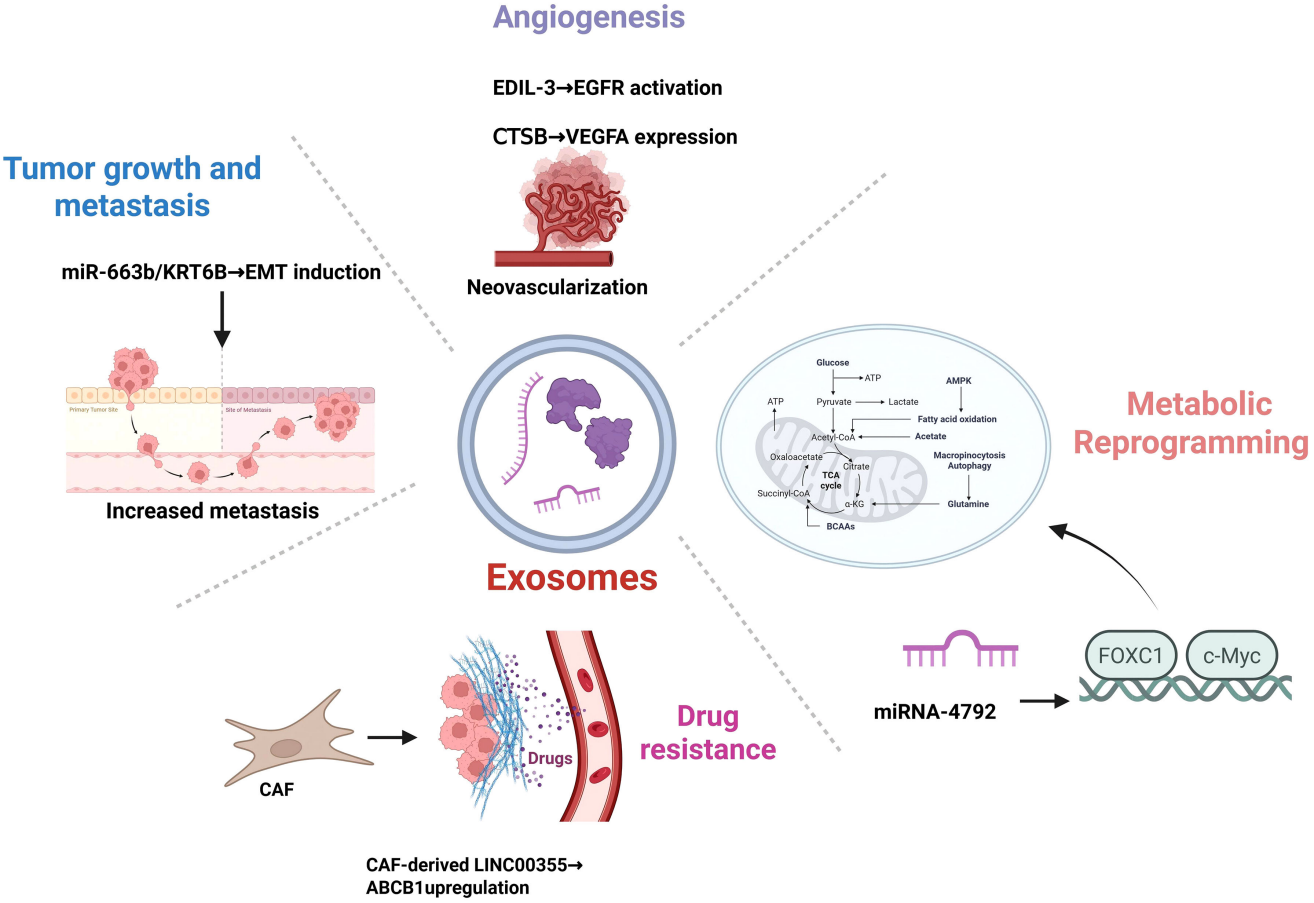
Exosomal regulation of BCa progression. This schematic summarizes the major exosome-mediated mechanisms involved in BCa progression across four functional modules: tumor growth and metastasis, angiogenesis, drug resistance, and metabolic reprogramming. In the tumor growth and metastasis module, exosomal miRNAs, lncRNAs, and proteins, including miR-663b, RMRP, LUCAT1, KRT6B, EDIL-3, and CTSB, promote malignant phenotypes by enhancing tumor cell proliferation, migration, invasion, EMT, and metastatic dissemination. In the angiogenesis module, exosomal signaling contributes to vascular remodeling and neovascularization, partly through activation of the EGFR/VEGFA-related pathway. In the drug resistance module, CAF-derived exosomal signaling upregulates ABCB1 and LINC00355 expression, thereby conferring enhanced chemoresistance to cisplatin and gemcitabine. In the metabolic reprogramming module, exosomal miR-4792 regulates the FOXC1/c-Myc axis, contributing to altered tumor cell metabolism.

Emerging evidence indicates that exosomes contribute to BCa progression by transferring oncogenic, pro-metastatic, metabolic, and immunomodulatory signals to neighboring tumor, stromal, endothelial, and immune cells (51). Exosomal cargoes can also mirror molecular features of the parental tumor, supporting their potential use as non-invasive biomarkers for diagnosis, prognosis, and disease monitoring (25). In fact, their molecular cargo can mirror the genetic and phenotypic attributes of the originating tumor, making them valuable for early detection. Their presence in body fluids enables the possibility of non-invasive liquid biopsies, offering a promising approach for detecting and monitoring early disease progression (52). Conversely, exosomes are being explored as therapeutic carriers that may deliver drugs or nucleic acids to cancer cells with improved biocompatibility (53). The following sections summarize the major functional roles of exosomes in BCa while indicating whether the supporting evidence is in the preclinical stage (**Figure 2**).

### 3.1 A cargo-context-recipient framework for interpreting BCa exosome biology

To address the context-dependent and sometimes divergent effects of exosomes, we propose a cargo-context-recipient framework. In this model, cargo refers to the molecular contents carried by exosomes, including miRNAs, lncRNAs, circRNAs, proteins, lipids, metabolites, and surface ligands. Context refers to the biological and therapeutic conditions under which vesicles are released, including tumor stage, cellular origin, hypoxia, inflammation, metabolic stress, treatment exposure, and the immune state of the TME. Recipient refers to the target cell and its functional state, such as malignant epithelial cells, endothelial cells, macrophages, dendritic cells, T cells, NK cells, fibroblasts, or stromal cells. This framework improves interpretation by emphasizing that exosomal effects are not determined by cargo alone. The same cargo class may promote tumor growth, immune suppression, or therapeutic sensitivity depending on the source cell, microenvironmental context, and recipient-cell program.

**Figure 3:**
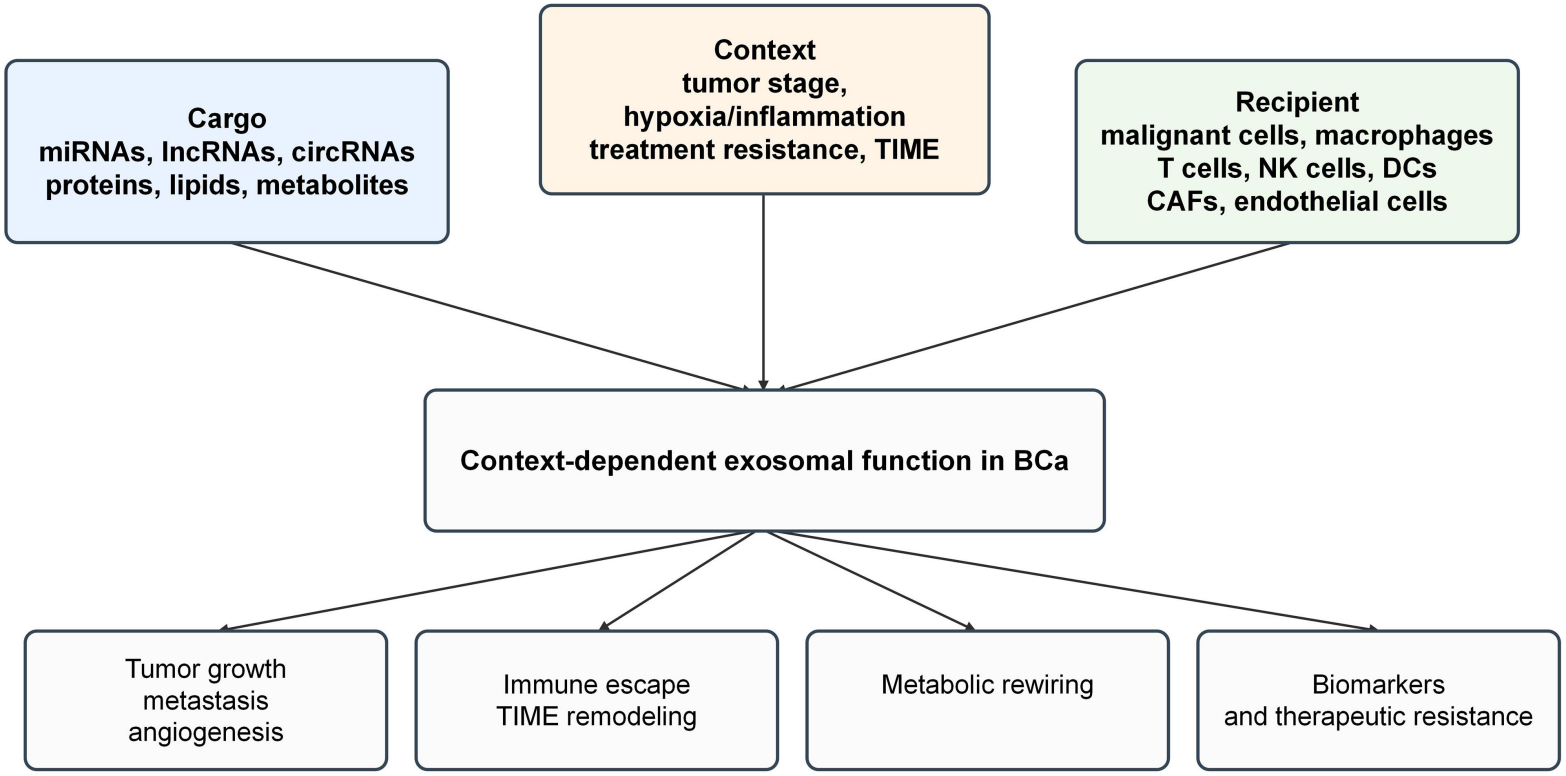
Cargo–context–recipient framework for interpreting context-dependent exosomal functions in bladder cancer. Exosomal functions in bladder cancer (BCa) are determined by the integrated effects of three key dimensions: the molecular cargo carried by exosomes, the biological context in which exosomes are released, and the functional state of recipient cells. Exosomal cargoes, including miRNAs, lncRNAs, circRNAs, proteins, lipids, and metabolites, may exert distinct biological effects depending on tumor stage, hypoxia, inflammation, treatment resistance, and the tumor immune microenvironment (TIME). Recipient cells, including malignant cells, macrophages, T cells, natural killer (NK) cells, dendritic cells (DCs), cancer-associated fibroblasts (CAFs), and endothelial cells, further shape the final biological outcome of exosome-mediated communication. This cargo–context–recipient model helps explain how exosomes contribute to tumor growth, metastasis, angiogenesis, immune escape, TIME remodeling, metabolic rewiring, biomarker development, and therapeutic resistance in BCa. BCa, bladder cancer; TIME, tumor immune microenvironment; NK cells, natural killer cells; DCs, dendritic cells; CAFs, cancer-associated fibroblasts.

Within this framework, BCa-associated exosomes should be interpreted as context-dependent signaling units rather than isolated vesicular biomarkers. This perspective helps reconcile discrepancies among studies and supports more rigorous experimental design: cargo profiling should be linked to donor-cell origin, disease stage, treatment condition, recipient-cell phenotype, and functional validation. It also provides a practical basis for biomarker development, because multidimensional signatures that integrate cargo, context, and recipient-cell information are likely to outperform single-cargo markers (**Figure 3**).

### 3.2 Tumor growth and metastasis

When exosomes transport pro-tumor factors, they facilitate the proliferation of tumor cells and advance the cell cycle (54). Additionally, the active components contained within these exosomes can inhibit cell apoptosis, therefore extending the survival of tumor cells and promoting both tumor growth and metastasis (27, 55). These effects are mediated by exosomal cargoes, including miRNAs, lncRNAs, proteins, and lipids, which regulate multiple signaling pathways (56, 57). LncRNAs can be selectively incorporated into exosomes and transferred to recipient cells, where they regulate tumor biology (58, 59). For example, exosomal lnc-TAF12-2:1 was upregulated in urinary exosomes, tumor tissues, and BCa cells. Its downregulation impaired BCa cell proliferation and migration, induced G0/G1 cell-cycle arrest, and increased apoptosis. Mechanistically, lnc-TAF12-2:1 competed with miR-7847-3p and participated in the miR-7847-3p/ASB12 axis to promote tumorigenesis (60). Exosomal lncRNA RMRP was associated with tumor progression, migration, and invasion through the miR-206/G6PD axis *in vitro* and *in vivo*, suggesting that the RMRP/miR-206/G6PD axis might be a potential therapeutic target (61). Ostenfeld et al. showed that silencing Rab27a and Rab27b reduced miR-23b and miR-921 levels in BCa-derived exosomes and decreased cell invasion, indicating that exosome-mediated miRNA transport could regulate metastatic behavior (62). Exosomal miR-4644, which is increased in the plasma of patients with BCa, promotes tumor growth by downregulating the tumor suppressor UbiA prenyltransferase domain containing 1 (UBIAD1) (63). Exosomal miR-663b has also been linked to epithelial-mesenchymal transition (EMT) through targeting ERF, a regulator of differentiation and proliferation (64).

In addition to RNAs, exosomes transport proteins that contribute to BCa pathogenesis. KRT6B in BCa-derived exosomes promoted EMT, invasion, and metastasis (65). EDIL-3/Del1 released by bladder tumor cells through exosomes enhanced tumor progression by facilitating cell adhesion and migration (66). Similarly, exosomal karyopherin alpha 2 (KPNA2) derived from cancer-associated fibroblasts (CAFs) interacts with kinesin family member C1 (KIFC1) to promote BCa progression (67). These findings support the role of exosomal proteins as functional drivers of BCa biology and potential therapeutic targets (**Table 1**).

**Table 1:** Functional roles of different exosomal cargoes in BCa progression.

Cargo	Exosome source	Mechanisms	Biological function
miR-663b	BCa cells	Target ERF; Promote EMT	Proliferation and Invasion
miR-4792	BCa cells	FOXC1/c-Myc axis	Metabolic reprogramming (Warburg effect)
miR-21-5p	BCa cells	Target PTEN	Proliferation and Metastasis
lnc-TAF12-2:1	BCa cells	Upregulate WNT8B	Proliferation and Invasion
RMRP	BCa cells	Target miR-206/TAC1 axis	Proliferation and Invasion
LUCAT1	BCa cells	Target miR-181a-5p/SMAD2 axis	Proliferation and Invasion
LINC00355	CAFs	Upregulate ABCB1	Chemoresistance (Cisplatin/Gemcitabine)
KRT6B	BCa cells	Induce EMT	Proliferation and Invasion
EDIL-3	BCa cells/Endothelial	Activate EGFR signaling	Angiogenesis and Progression
CTSB	BCa cells/Endothelial	TPX2-AURKA-PI3K-AKT pathway	Neovascularization (VEGFA expression)
KPNA2	Cancer-associated fibroblasts (CAFs)	Interacted with KIFC1	Proliferation and Invasion

### 3.3 Angiogenesis

Tumor growth and metastatic progression depend on an angiogenic supply of oxygen and nutrients. Without neovascularization, tumor expansion is biologically constrained (68). Although vascular endothelial growth factor (VEGF) is a major driver of angiogenesis, exosomal signaling also contributes to vascular remodeling in the BCa microenvironment (69, 70). BCa cell-derived exosomal GFAT1 can induce endothelial metabolic reprogramming through the hexosamine biosynthesis pathway, leading to SerRS O-GlcNAcylation (71). Furthermore, research by Carla J et al. indicated that exosomes from high-grade BCa cells actively stimulated endothelial migration and vessel formation. This was partially mediated by exosomal EDIL-3, which triggered epidermal growth factor receptor (EGFR) signaling to enhance cellular motility. Additionally, serum-derived exosomes from BCa patients exhibited elevated levels of cathepsin B (CTSB) (66). As demonstrated by Li et al, the uptake of EV-encapsulated CTSB activated a TPX2-dependent AURKA-PI3K-AKT phosphorylation cascade, ultimately upregulating VEGFA expression and catalyzing tumor-associated angiogenesis (72).

### 3.4 Metabolic reprogramming and drug resistance

Metabolism encompasses a complex network of biochemical reactions that transform nutrients into small molecules known as metabolites (73). However, the metabolic processes of tumors differ significantly from those of normal cells. As first noted by Otto Warburg, cancer cells exhibit a preference for glycolysis in the cytoplasm, even when oxygen is available. This phenomenon is referred to as the Warburg effect or aerobic glycolysis (74, 75). This shift from oxidative phosphorylation to glycolysis is shaped by genetic alterations and microenvironmental pressures (76). Enhanced glycolysis and reduced mitochondrial oxidative phosphorylation can support malignant growth, survival, and invasion (77). This mechanism supports the proliferation and survival of malignant tumors and significantly increases the invasive capabilities of cancer cells. For example, Wu et al. provided the first evidence that exosome-mediated delivery of miR-4792 significantly influenced BCa progression by downregulating Forkhead Box C1 (FOXC1) and c-Myc, thereby promoting aerobic glycolysis and lactic acid production. These effects ultimately promoted cell proliferation and tumor growth (78).

Gemcitabine (GEM)-based chemotherapy remains an important treatment option for advanced BCa (79), but acquired resistance limits its durability. Chemoresistance can result from genetic and epigenetic alterations, drug efflux, altered drug metabolism, DNA damage repair, survival pathway activation, and TME-mediated protection (80, 81). Exosomes could transfer resistance-associated signals to chemotherapy-sensitive cells (82). In BCa, chemotherapy enriched stem-like cells and upregulated lung cancer-associated transcript 1 (LUCAT1), which promoted chemoresistance by enhancing stemness. Exosomes from BCa stem cells delivered LUCAT1 to recipient cells, stabilizing high mobility group A1 (HMGA1) mRNA through an m6A-dependent mechanism involving IGF2BP2. These findings suggest that exosomal LUCAT1 may function as both a biomarker and a therapeutic target for chemoresistant BCa (83).

CAFs also secreted exosomal miR-146a-5p in BCa. Mechanistically, miR-146a-5p increased Sushi, von Willebrand factor type A, EGF and pentraxin domain containing 1 (SVEP1) expression in CAFs by recruiting the transcription factor YY1. In BCa cells, CAF-derived miR-146a-5p targeted ARID1A and PRKAA2, thereby promoting stemness and chemoresistance (84).

Cisplatin resistance presents a significant challenge in the treatment of BCa (80). CAF-derived exosomes can enhance chemotherapy resistance in several tumors by transferring bioactive molecules (85). In BCa, CAF-derived exosomal LINC00355 promoted cisplatin resistance by sponging miR-34b-5p and upregulating ABCB1. Silencing ABCB1 or overexpressing miR-34b-5p reversed this effect, highlighting the functional role of LINC00355 in drug resistance (86). CAF-derived exosomes could also deliver miR-148b-3p to BCa cells, promoting EMT, metastasis, and resistance to paclitaxel and doxorubicin. Restoration of PTEN counteracted EMT, metastasis, and chemoresistance, thereby opposing the tumor-promoting effects of miR-148b-3p through the Wnt/beta-catenin pathway (87).

### 3.5 Exosomes as non-invasive diagnostic tools in BCa

Research on exosomal biomarkers in BCa has largely focused on urine, a biofluid that directly contacts the urothelium and is enriched in bladder-derived cargo (22). Urinary exosomal miRNAs are the most extensively investigated biomarker class. Numerous studies have reported altered exosomal miRNA signatures with potential diagnostic or staging value (88). Andreu et al. (89) performed qRT–PCR on first-morning preoperative urine specimens from 34 individuals with bladder cancer (18 high-grade and 16 low-grade urothelial carcinomas) and 9 healthy controls. Among patients with high-grade disease, miR-375 was the only miRNA that showed a statistically significant decrease relative to controls, whereas in low-grade tumors, miR-146a was significantly enriched in urinary exosomes. Furthermore, exosome-derived miRNAs facilitated the identification of early-stage malignancy, including NMIBC cases that were often missed by conventional urine cytology via conventional urine cytology. Notable examples include miR-93-5p and miR-375, which were enriched in the urinary exosomes of BCa patients and correlated with aggressive, high-grade tumors and unfavorable outcomes (90, 91). Monitoring for disease recurrence might also be improved through these biomarkers; miR-194-5p and miR-155 showed elevated expression in the urinary exosomes of patients experiencing relapse (25, 92). Additionally, Matsuzaki et al. (93) used miRNA microarrays to profile serial urine samples obtained from six patients with urothelial carcinoma (from admission until surgery) and three healthy volunteers, followed by validation in an independent cohort of 36 bladder cancer patients and 24 controls. Five miRNAs were consistently upregulated in urinary exosomes from the cancer group compared with controls, with miR-21-5p emerging as the most informative biomarker for urothelial carcinoma detection (area under the curve (AUC) = 0.900; sensitivity, 75.0%; specificity 95.8%). Nonetheless, these results warrant confirmation in larger, rigorously designed validation studies.

Beyond miRNA profiles, exosomal lncRNAs may expand the diagnostic repertoire. Specific lncRNAs, including SPRY4-IT1, MALAT1, and PCAT-1, show differential expression in BCa-derived vesicles (94).When utilized in combination, these biomarkers yield a superior AUC of 0.813, showing potential to outperform conventional cytology in preliminary studies (94). Multiplex panels that integrate several urinary exosomal biomarkers appear to further enhance diagnostic performance. A panel of seven glucocorticoid-related lncRNAs (GR-lncRNAs) including AL390728.6, AL355353.1, AC011477.2, AL357033.4, AC073210.3, AC105942.1, and FRMD6-AS2 was identified to construct a prognostic model with strong predictive performance. Patients classified as high risk exhibited markedly poorer survival and distinct immune landscapes, suggesting important implications for individualized therapeutic strategies (95). Consequently, the integration of lncRNA profiling represents a potent strategy for enhancing the non-invasive molecular characterization of bladder malignancies.

Extensive investigations have underscored the remarkable diagnostic potency of exosomal molecules in BCa. For instance, exosomal CEACAM1 demonstrated a high AUC of 0.907. Similarly, exosomal miR-96-5p yielded an AUC of 0.87, characterized by 82.4% sensitivity and 91.8% specificity (96). To further enhance diagnostic accuracy, researchers have developed multi-biomarker panels; a combination of exosomal UCA1-201, UCA1-203, MALAT1, and LINC00355 achieved a superior AUC of 0.96 (97). Other significant markers include exosomal CA9, TERC, and various lncRNA signatures (e.g., ANRIL and GAS5), which provided diverse platforms for non-invasive screening (98–101). Beyond initial detection, exosomes serve as pivotal indicators for prognostic stratification.

## 4 Exosomes facilitate BCa development via crosstalk in the TME

**Figure 4:**
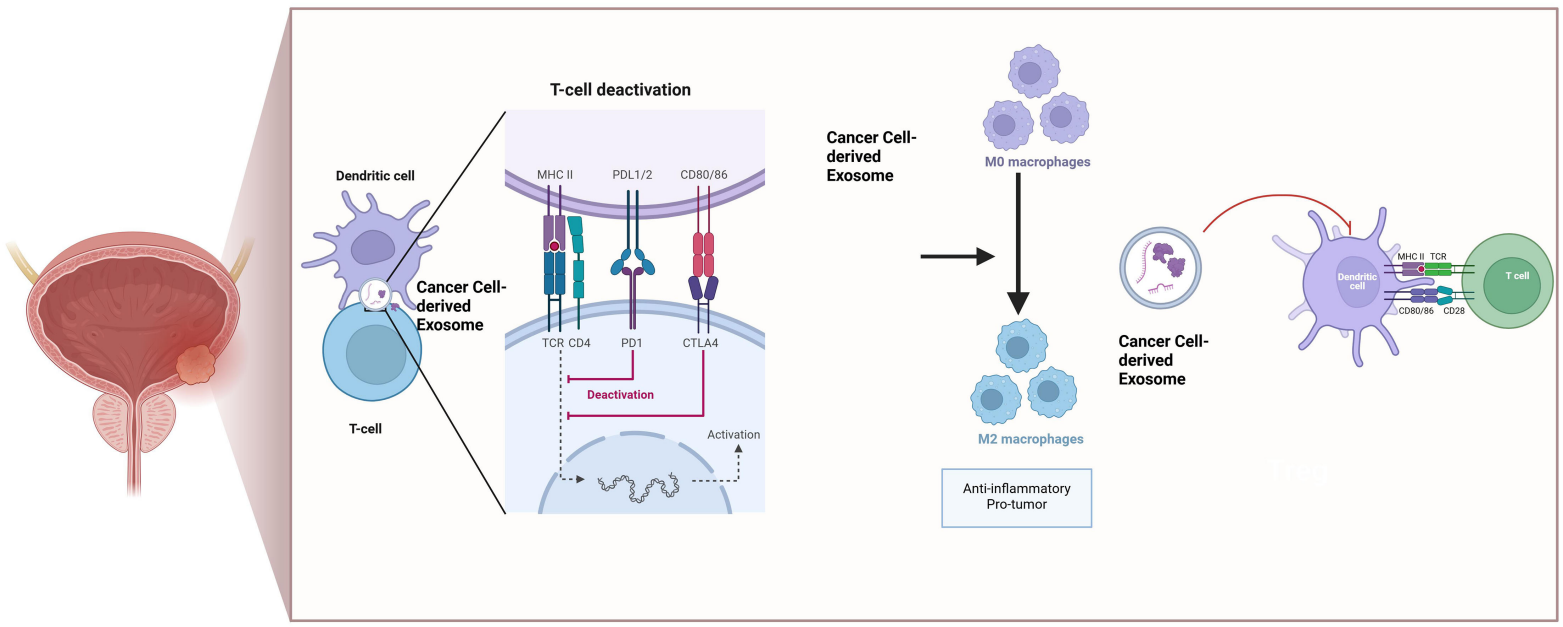
Exosome-mediated regulation in the TME. BCa-derived exosomes reshape the tumor immune microenvironment (TIME) by acting on multiple immune compartments. Tumor-derived exosomes may promote T-cell exhaustion through programmed death-ligand 1 (PD-L1)/programmed cell death protein 1 (PD-1) signaling, leading to reduced cytotoxicity and increased expression of exhaustion markers. Exosomes also contribute to macrophage polarization by favoring the transition from pro-inflammatory M1-like phenotypes toward immunosuppressive M2-like macrophages, partly through transforming growth factor beta (TGF-beta)-related signaling and exosomal miRNA-mediated regulation. In dendritic cells (DCs), tumor-derived exosomes may reduce major histocompatibility complex class II (MHC-II) expression, impair antigen processing, and weaken T-cell activation. Together with myeloid-derived suppressor cell (MDSC) accumulation and natural killer (NK) cell dysfunction, these mechanisms establish an immunosuppressive landscape that facilitates tumor immune evasion. BCa, bladder cancer; TIME, tumor immune microenvironment; TME, tumor microenvironment; ECM, extracellular matrix; PD-L1, programmed death-ligand 1; PD-1, programmed cell death protein 1; TGF-beta, transforming growth factor beta; IL-10, interleukin-10; DC, dendritic cell; MHC-II, major histocompatibility complex class II; MDSC, myeloid-derived suppressor cell; NK cell, natural killer cell; M1, pro-inflammatory macrophage phenotype; M2, anti-inflammatory or immunosuppressive macrophage phenotype; CD206, cluster of differentiation 206; Arg1, arginase 1; TNF-alpha, tumor necrosis factor alpha; GZMB, granzyme B; IFN-gamma, interferon gamma; TLAG-3, T-cell lymphocyte activation gene 3.

BCa is not just a passive mass of cancer cells; it consists of a diverse array of immune cell and non-immune cell types that engage in complex interactions, collectively forming the TIME (10). The investigation of the TIME and the reorganization of the cellular regulatory network have significantly advanced the identification of therapeutic targets in cancer biology. The TIME plays a vital role in the survival and proliferation of tumors (13). As tumors progress, the TIME transitions from a state of immune surveillance to one characterized by immune suppression. Initially, immune cells within the TIME attempt to target and eliminate the tumor; however, as the tumor evolves, it alters the microenvironment to dampen these immune responses (102). Exosomes are central to this process, facilitating critical intercellular communication within the TIME (**Figure 4**).

With respect to immune modulation, BCa cell-derived exosomes promote macrophage polarization toward the M2 phenotype, thereby fostering an immunosuppressive TME that supports tumor growth. For example, the expression of ERα was positively associated with the formation of vascular mimicry (VM) and the infiltration of M2-type tumor-associated macrophages (TAMs). Mechanistic studies demonstrated that the exosomal miR-642a-5p, upregulated by ERα in BCa cells, reduces PTEN expression in macrophages by directly targeting the 3’ UTR of PTEN mRNA. These findings offered new insights for the development of innovative therapeutic strategies for BCa (103). Additionally, exosomal miR-21 released by BCa cells promoted the polarization of macrophages to the M2 phenotype, thereby creating an immunosuppressive TME that supported tumor growth. Exosomal miR-21 facilitated these processes by downregulating tumor suppressor genes like PTEN and PDCD4, activating the PI3K/Akt signaling pathway, and playing a role in carcinogenesis (104). Moreover, TAMs also secreted exosomes that play an essential role in BCa progression. Exosomal lncRNA HISLA was secreted by TAMs, which stabilized β-catenin expression by preventing its interaction with GSK3β, leading to enhanced EMT in BCa cells (105).

Besides TAMs, exosomes are also implicated in the modulation of other immune cells. Yang et al. revealed that exosome-derived hsa_circ_0085361 (circTRPS1) sponged miR-141-3p, thus influencing alterations in glutamine metabolism mediated by GLS1. Exosomes from BCa cells with circTRPS1 knocked down not only inhibited tumor growth but also prevented CD8^+^ T cell exhaustion, thereby reducing the malignant characteristics of BCa cells. These findings revealed that circTRPS1/miR141-3p/GLS1 serve as a novel therapeutic target for BCa (51). Moreover, exosomes derived from the urinary BCa cell line contributed to the dysfunction of natural killer (NK) cells by reducing their viability and inhibiting their cytotoxic effects on target cells. Additionally, these exosomes downregulated the expression of key functional receptors NKG2D, NKp30, and CD226 on NK cells, as well as the secretion of perforin and granzyme B (106). Understanding the specific mechanisms of these exosomes will aid in evaluating their potential as therapeutic targets, thereby boosting anti-tumor immunity and improving patient outcomes.

## 5 Exosome-based strategies for BCa treatment

Despite advancements in traditional treatment methods such as surgery and chemotherapy, the prognosis for BCa remains poor, with persistently high mortality rates (107). This underscores an urgent need for innovative therapies that are safe, effective, and precisely targeted. Exosomes serve as crucial messengers in intercellular communication, and their ability to transport biomolecules to recipient cells makes them highly attractive for drug delivery applications (108, 109). Although exosomes resemble liposomes in size, shape, and overall structure, they have a more complex lipid bilayer that contains diverse lipids, proteins, carbohydrates, internal cargoes, and surface-associated molecules. Although exosomes share several similarities with liposomes, they exhibit greater membrane curvature and asymmetry, which enhance their interactions with cell membranes (110). Additionally, the abundance of membrane proteins on exosomes provides greater potential for drug delivery. Compared to artificially synthesized nanocarriers, exosomes are derived from cells and can be more readily metabolized after drug delivery. Additionally, they minimize the risk of eliciting unwanted immune responses (108, 111). These favorable characteristics position exosomes as promising candidates for novel drug delivery systems, particularly in enhancing immune modulation and targeting specificity.

Exosome-mediated therapeutic interventions are currently undergoing intensive investigation across a spectrum of pathologies, with increasing attention to BCa. Emerging evidence underscores the pivotal role of exosomal miRNAs in orchestrating the molecular landscapes of BCa, particularly in modulating oncogenic pathways that drive tumor development, metastatic dissemination, and the acquisition of chemoresistance (112, 113). Pan et al. revealed that bladder cancer stem cells (BCSCs)-derived exosomes (CSCs-exo) acted as pivotal mediators in orchestrating a stable tumor microenvironment. Mechanistically, BCSC-derived exosomes delivered miR-105-5p to recipient cells, where it directly targeted and suppressed G-protein-coupled receptor 12 (GPR12). The oncogenic effects of the miR-105-5p/GPR12 axis were further validated *in vivo*, where miR-105-5p antagomirs successfully attenuated tumor growth. These results highlighted a novel signaling circuit in the BCSC niche, offering fresh targets for stem cell-oriented interventions (114). Exosomes derived from bone marrow mesenchymal stem cells (BMSCs) functioned as efficient conduits for the transport of lncRNA PTENP1 in suppressing BCa advancement. Exosomal PTENP1 significantly curtailed BCa cell proliferative, migratory, and invasive potential while promoting apoptosis. Mechanistically, PTENP1 acted as a molecular sponge for miR-17, thereby regulating the expression of SCARA5. Notably, exosomes harvested from PTENP1-overexpressing BMSCs neutralized the oncogenic effects induced by miR-17 enrichment or SCARA5 depletion. These *in vivo* and *in vitro* findings identify the PTENP1/miR-17/SCARA5 regulatory axis as a vital determinant of BCa progression and a promising therapeutic target (115). Exosomal miR-133b, generated via transfection-based loading in BCa cells, has demonstrated potent anti-cancer properties. Exosome-mediated delivery of this miRNA effectively suppressed BCa cell survival and promoted programmed cell death *in vitro*, alongside significant tumor growth inhibition in mouse models. The underlying therapeutic mechanism involved the positive regulation of DUSP1, indicating that the exosomal miR-133b/dual specificity phosphatase 1 (DUSP1) pathway represented a promising target for future clinical interventions in BCa (116). These findings furnish a robust conceptual framework for the development of next-generation exosome-based drug delivery strategies.

Beyond their endogenous roles, exosomes can be bioengineered to encapsulate exogenous RNA and protein payloads for site-specific therapeutic delivery. Within the realm of BCa research, these engineered exosomes have demonstrated significant clinical promise (117). For instance, Liu et al. revealed that miR-138-5p-loaded exosomes, derived from adipose-derived mesenchymal stem cells (ADSCs), could penetrate tumor tissue and effectively suppress xenograft growth (118). Similarly, mesenchymal stem cells (MSCs)-derived exosomal miR-139-5p has been identified as a potent regulator capable of blunting the oncogenic progression of BCa. These integrated data corroborated the tumor-antagonizing role of exosomal miR-139-5p originating from MSCs. Such results advocated for the development of exosome-mediated miRNA delivery systems as a promising and innovative modality for tackling BCa malignancy (119). Moreover, research by Shan et al. elucidated that CAFs-derived exosomes, enriched with miR-148b-3p, facilitated BCa advancement. This oncogenic effect was mediated through the suppression of PTEN and subsequent stimulation of the Wnt/β-catenin signaling axis, which drove EMT, metastatic spread, and resistance to chemotherapy. Consequently, pharmacological inhibition of exosomal miR-148b-3p has emerged as a viable approach to counteract chemoresistance (87). Conversely, Li et al. reported that exosome-mediated transfer of miR-375-3p exerted antitumor effects by antagonizing the Wnt/β-catenin pathway, suggesting its potential utility as a therapeutic strategy for restricting BCa progression (120). Furthermore, exosomes provide a promising platform for drug delivery because their membranes protect therapeutic cargoes from degradative environments. This property enables the targeted delivery of small-molecule drugs, such as paclitaxel and gemcitabine, which are being actively explored to improve treatment outcomes. While some pioneering studies on exosome-mediated delivery were initially conducted in other malignancies, such as prostate and pancreatic cancers (121, 122), their application in BCa is rapidly advancing. For instance, recent studies have demonstrated that exosomes could potentially overcome current drug resistance and improve patient outcomes (83). Ultimately, these exosome-centered innovations provide promising prospects for enhancing the therapeutic efficacy and clinical prognosis of BCa patients.

Traditional intracellular RNA delivery, typically mediated by synthetic nanoparticles or viral vectors, was often hampered by significant cytotoxic side effects (123). As a superior biocompatible alternative, exosomes have emerged as ideal therapeutic vehicles (124). By engineering exosomes to encapsulate specific signaling modulators, researchers could effectively reprogram the malignant phenotype of tumor cells (125, 126). For instance, Zhou et al. developed a sophisticated molecular device by fusing the exosomal marker CD63 with an RNA-binding protein that possesses high affinity for AU-rich elements (AREs). Concurrent with this, artificial circular RNAs (acircRNAs) were designed using aptamer-based designs to simultaneously antagonize β-catenin and NF-κB signaling in bladder cancer. By integrating ARE motifs into the acircRNA sequence, these therapeutic molecules were selectively partitioned into exosomes. These acircRNA-loaded exosomes possessed potent anti-tumor properties, exhibiting superior suppressive efficiency, thereby providing a potential strategy for BCa treatment (127). Exosomes harvested from HEK293 and MSCs have demonstrated significant efficacy as specialized biological vehicles for transporting polo-like kinase 1 (PLK-1) small interfering ribonucleic acid (siRNA) into BCa cell populations. This exosome-mediated delivery facilitated highly selective gene silencing, effectively downregulating PLK-1 expression *in vitro*. Given their biocompatibility and inherent targeting capabilities, these exosomes represented a compelling platform for the advancement of localized intravesical therapies, offering a sophisticated approach to precision oncology in the bladder (128).

## 6 Exosomes based immunotherapy in BCa

Exosomes have emerged as promising tools in the clinical oncology landscape, functioning as versatile EVs that bridge the gap between non-invasive diagnostics and targeted molecular therapeutics (117). Their capacity to encapsulate diverse bioactive cargoes and faithfully recapitulate the phenotypic characteristics of their parental cells has positioned them as premier candidates for liquid biopsy-based biomarkers in detecting, prognosticating, and monitoring treatment responses across various malignancies, including pulmonary, mammary, gastric, and thyroid carcinomas (19, 129). Data retrieved from ClinicalTrials.gov have highlighted this momentum, with nearly 90 trials exploring exosome-based applications in cancer; notably, 93% of these investigations prioritize diagnostic and monitoring utilities over direct interventional roles. This clinical preference stems from the inherent advantages of exosomes over traditional tissue biopsies, as they can be isolated from readily accessible biofluids such as blood, urine, or saliva, facilitating real-time assessment of disease dynamics. Beyond diagnostics, exosomes are increasingly being evaluated as therapeutic platforms in colorectal, pancreatic, and hematological cancers like acute myeloid leukemia (AML). Although it is still a nascent field, exosome-mediated immunotherapy is gaining traction, with registered trials (e.g. NCT03985696 (130), and NCT05427227 (131)) evaluating immune biomarkers and therapeutic efficacy in hepatocellular and renal cell carcinomas (26).

In the field of BCa, exosome-related immunotherapy research is still largely confined to the preclinical stage. Current clinical research is predominantly biomarker-centric, focusing on urinary exosomal glycosylation patterns (NCT04960956), lncRNA-ELNAT1 expression (NCT05270174), and RNA profiles (NCT06193941) (132–134). However, the therapeutic horizon is expanding with trials like NCT05559177, which explores a personalized chimeric exosome vaccine for metastatic BCa. The integration of exosome technology with established immunotherapies, such as immune checkpoint inhibitors and adoptive cell therapy, represents a potent synergistic strategy (135). Exosomes can be bioengineered to deliver immunostimulatory molecules that disrupt the PD-1/PD-L1 signaling axis or transport tumor antigens to optimize DC priming and T-cell expansion, thereby overcoming the immunosuppressive mechanisms often encountered in BCa (104, 136). Although immunotherapy has a long history in bladder cancer, as exemplified by conventional Bacillus Calmette-Guérin (BCG) therapy and numerous registered trials involving autologous cellular interventions, no exosome-based immunotherapeutic strategy has yet reached the stage of regulatory approval for BCa.

Tumor-derived exosomes (TEXs) are emerging as sophisticated platforms for cell-free vaccine development, owing to their inherent ability to facilitate antigen presentation (60). Within this biological framework, non-coding RNAs (ncRNAs) function as pivotal orchestrators of immune cell ontogeny, maturation, and effector activities. Consequently, the strategic modification of TEXs with specialized ncRNAs represents a transformative approach for augmenting immunotherapy efficacy in BCa (135, 137). The rising paradigm of personalized medicine offers a promising trajectory, where patient-specific exosomes can be modified into personalized vaccines that reflect a unique tumor profile, thereby stimulating a tailored immune response (138). Although several exosome-based therapeutic strategies have been investigated in other malignancies, bladder cancer-specific evidence remains limited. Therefore, findings extrapolated from other tumor types should be interpreted cautiously until they are validated in BCa-specific models and clinical studies. Ultimately, while exosome-based immunotherapy for BCa faces significant translational hurdles, continued preclinical investigation into immune modulation pathways and TME interactions is essential to accelerate these innovative vesicles from laboratory research to standard clinical care.

## 7 Conclusions and perspectives

Accumulating evidence supports the potential of exosomes as biomarkers and drug-delivery platforms, with encouraging results in preclinical studies (139). Based on the current literature, three key messages emerge. First, the biological impact of BCa-derived exosomes is strongly influenced by tumor stage and the functional state of recipient cells within the TME. Second, urinary exosomes provide a non-invasive window into bladder pathology and may capture aspects of tumor heterogeneity that are difficult to assess using single-site tissue biopsy. Third, natural exosomes are biocompatible but often lack the potency, reproducibility, and targeting precision required for clinical efficacy, making rational bioengineering and biomimetic approaches important future directions.

To move beyond descriptive cataloguing of exosomal cargoes, BCa-associated exosomes should be interpreted as context-dependent signaling units. In the cargo-context-recipient framework, exosomal function is determined by the interaction among three variables: the molecular cargo carried by the vesicle, the biological or therapeutic context in which the vesicle is released, and the functional state of the recipient cell. This model helps explain why similar cargo classes can have divergent effects across BCa stages, treatment settings, and immune states. Tumor- and stromal-cell-derived exosomes can shift cancer cells toward more invasive, metabolically rewired, and drug-resistant phenotypes; activate endothelial cells and angiogenesis; and remodel immune compartments by promoting T-cell exhaustion, NK-cell dysfunction, M2 macrophage polarization, dendritic-cell impairment, and possibly MDSC enrichment (140). Thus, exosomes act as dynamic organizers of tumor ecological adaptation, linking tumor-intrinsic evolution with stromal remodeling, immune escape, and therapeutic resistance.

This integrative model has several implications for future research. Biomarker studies should move from single-cargo detection toward multidimensional signatures that combine cargo composition, vesicle source, tumor stage, and immune context. Mechanistic studies should prioritize causal validation of exosome-mediated communication networks in clinically relevant BCa models rather than relying only on correlative cargo profiling. Therapeutic strategies should not only engineer exosomes as delivery vehicles but also target context-specific processes such as cargo loading, vesicle release, recipient-cell uptake, and immune reprogramming. Such a framework may help transform BCa exosome research into a mechanism-guided platform for biomarker development, patient stratification, and rational therapeutic design (140).

Despite promising preclinical results, translation from bench to bedside is hindered by several bottlenecks. A major problem is the lack of standardized isolation, purification, and characterization methods. Differences among ultracentrifugation, size-exclusion chromatography, precipitation, immunoaffinity capture, and microfluidic approaches can lead to variability in exosome purity, yield, and cargo integrity. Therapeutic applications also face a dosage dilemma: the optimal concentration must maximize antitumor activity while minimizing off-target toxicity, unwanted immune suppression, or pro-tumor signaling. These challenges have motivated the development of engineered exosomes with improved targeting, cargo loading, and functional control (141). Moreover, combining exosomes with other nanomaterials, such as liposomes and polymeric nanoparticles, can result in synergistic therapeutic effects, thereby increasing the overall efficacy of the treatment (142). The development of bio-inspired or biomimetic exosomes represents a significant advancement, offering innovative strategies for clinical applications in exosome-based drug delivery systems (143, 144). Despite the considerable advantages that engineered exosomes present over natural ones, several challenges remain in their clinical translation.

Firstly, there is currently no consensus on standardized methods for isolating, quantifying, and analyzing engineered exosomes derived from complex biological samples, such as blood, tissue, and urine (145). This lack of standardization complicates the comparison of results across different studies and hinders advancement in the field (146). Secondly, accurately quantifying exosomes and their cargo remains a challenge (147). Options for quantification, including determining the number of exosomes, their protein content, the ratio of both, or employing classical monoclonal antibody microarray-based surface profiling, have yet to be fully clarified and standardized (148). Finally, given that exosomes derived from different sources may have distinct functions and compositions, identifying the most suitable and available exosomes for specific applications poses another significant hurdle (149). Addressing these issues is vital for the successful development of exosome-based drug delivery systems in clinical settings.

### 7.1 Limitations

Several limitations should be acknowledged. Firstly, most available evidence regarding exosomes in BCa comes from retrospective studies, *in vitro* experiments, or animal models. Clinical evidence supporting exosome-based therapeutics in BCa remains very limited, and many proposed strategies require validation in well-designed clinical trials. Second, substantial heterogeneity exists in exosome isolation, purification, quantification, and characterization across studies, which complicates comparison and limits reproducibility. Third, many biomarker studies involve small cohorts and lack independent external validation, reducing their generalizability and clinical applicability. Fourth, some therapeutic concepts discussed in this review are extrapolated from hepatocellular carcinoma, colorectal cancer, pancreatic cancer, renal cell carcinoma, or other malignancies rather than from BCa-specific evidence. Such findings should be interpreted cautiously until validated in BCa-specific models and clinical studies. Finally, exosome biology and engineered exosome therapeutics are rapidly evolving, and several conclusions may require revision as new mechanistic data and clinical trial outcomes become available.

Looking ahead, engineered exosomes remain a promising but still investigational platform. Immune-cell-derived exosomes, nanomaterial-encapsulated vesicles, and aptamer-coated exosomes may eventually be integrated with external ultrasound irradiation or other image-guided approaches to improve tumor targeting (150, 151). Such combinations could enhance treatment specificity and efficacy while reducing systemic toxicity (152–154). However, their clinical translation in BCa will require rigorous validation of biodistribution, reproducibility, manufacturing scalability, immune effects, and long-term safety.
