# Supplementary material for: Decoding the role of exosomes in bladder cancer: focusing on tumor progression and immune microenvironment modulation
Source: Front Oncol. 2026 Jul 31;16:1821922. doi: 10.3389/fonc.2026.1821922 (PMC13474255; doi:10.3389/fonc.2026.1821922)
Supplement: Supplementary file 1 [file Table1.docx]

**Supplementary Table 1. Full literature search strategy**

| Database | Exact search string | Filters and search date |
| --- | --- | --- |
| PubMed | ("bladder cancer"[Title/Abstract] OR "urothelial carcinoma"[Title/Abstract]) AND (exosome*[Title/Abstract] OR "extracellular vesicle"[Title/Abstract] OR "extracellular vesicles"[Title/Abstract]) AND ("tumor microenvironment"[Title/Abstract] OR "immune microenvironment"[Title/Abstract] OR biomarker*[Title/Abstract] OR "drug delivery"[Title/Abstract] OR immunotherap*[Title/Abstract] OR microRNA[Title/Abstract] OR miRNA[Title/Abstract] OR lncRNA[Title/Abstract] OR chemoresistance[Title/Abstract] OR angiogenesis[Title/Abstract]) | English language; original research, reviews, and clinically relevant studies; final search date: January 5, 2026 |
| Web of Science | TS= (("bladder cancer" OR "urothelial carcinoma") AND (exosome* OR "extracellular vesicle*" OR EVs) AND ("tumor microenvironment" OR "immune microenvironment" OR biomarker* OR "drug delivery" OR immunotherap* OR microRNA OR miRNA OR lncRNA OR chemoresistance OR angiogenesis)) | Document types: article or review; English language; final search date: January 5, 2026 |
| Scopus | TITLE-ABS-KEY (("bladder cancer" OR "urothelial carcinoma") AND (exosome* OR "extracellular vesicle*" OR EVs) AND ("tumor microenvironment" OR "immune microenvironment" OR biomarker* OR "drug delivery" OR immunotherap* OR microRNA OR miRNA OR lncRNA OR chemoresistance OR angiogenesis)) | Language: English; articles and reviews; final search date: January 5, 2026 |
